# Improving medication adherence in type 2 diabetes: strategies for better clinical and economic outcomes

**DOI:** 10.1007/s00125-025-06617-x

**Published:** 2025-11-28

**Authors:** Patrick J. Highton, Mark P. Funnell, Pankaj Gupta, Francesco Zaccardi, Lee-Ling Lim, Samuel Seidu, Kamlesh Khunti

**Affiliations:** 1 Diabetes Research Centre, University of Leicester, Leicester General Hospital, Leicester, UK; 2 National Institute for Health and Care Research Applied Research Collaboration East Midlands, Leicester, UK; 3 Department of Cardiovascular Health Sciences, University of Leicester, Leicester, UK; 4 Leicester Real World Evidence Unit, Diabetes Research Centre, University of Leicester, Leicester General Hospital, Leicester, UK; 5 Department of Medicine, Faculty of Medicine, Universiti Malaya, Kuala Lumpur, Malaysia; 6 Department of Research, Development and Innovation, Universiti Malaya Medical Centre, Kuala Lumpur, Malaysia; 7 Department of Medicine and Therapeutics, The Chinese University of Hong Kong, Hong Kong, Hong Kong SAR China; 8 Asia Diabetes Foundation, Hong Kong, Hong Kong SAR China; 9 Baker Heart and Diabetes Institute, Melbourne, VIC Australia

**Keywords:** Glycaemic management, Healthcare outcomes, Interventions, Medication adherence, Multimorbidity, Review, Type 2 diabetes mellitus

## Abstract

Medication adherence is critical for optimal glycaemic management and the prevention of complications in type 2 diabetes mellitus. Despite its importance, non-adherence remains a prevalent issue, with global estimates suggesting that approximately 38% of people with type 2 diabetes do not take their medications as prescribed, although estimates vary widely due to a range of patient-related, socioeconomic, condition-related (e.g. chronicity, severity of comorbidities) and healthcare system factors. This review synthesises the current evidence on the prevalence of non-adherence in type 2 diabetes, as well as risk factors and clinical and economic consequences, and evaluates interventions designed to improve adherence in this population. Medication non-adherence is associated with increased HbA_1c_ levels and risks of micro- and macrovascular complications, hospitalisation and mortality, while also incurring substantial healthcare costs. Methods for assessing medication adherence range from subjective self-report tools to objective measures such as biochemical testing, which are gaining prominence in both research and clinical practice. Numerous interventions to increase medication adherence have been investigated, including educational, technological, pharmacist-led, behavioural and financial strategies and fixed-dose combination (FDC) therapies. While interventions such as pharmacist-led care and FDC therapies are beneficial, outcomes from technology-based and motivational strategies remain mixed. Shared decision making and behavioural interventions, such as cognitive behavioural therapy, may provide additional benefits, particularly for individuals with multiple long-term conditions or psychological comorbidities. Multifaceted interventions tailored to individual barriers and sustained over time appear most effective. Given the significant impact of non-adherence on both patient outcomes and healthcare systems, future research is needed to design personalised, multicomponent interventions that are scalable and equitable across diverse healthcare settings.

**Graphical Abstract:**

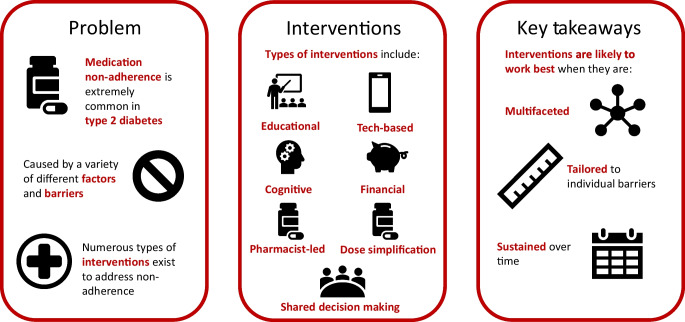

**Supplementary Information:**

The online version contains a slideset of the figures for download available at 10.1007/s00125-025-06617-x.

## Introduction

Adherence to prescribed treatment regimens is a cornerstone of the effective management of chronic health conditions such as diabetes and CVD. WHO defines adherence as ‘the extent to which a person’s behaviour – taking medication, following a diet, and/or executing lifestyle changes, corresponds with agreed recommendations from a health care provider’ [1]. The WHO also reports that, among individuals with chronic conditions in developed countries, up to 50% of medications are not taken as prescribed [1]. However, although this figure is commonly cited, the true value may vary widely depending on the assessment period, specific medications, disease states and populations [2]. A more comprehensive meta-analysis of 328 studies that provided data on medication adherence found adherence rates (measured using various methodologies) to be 79% [3]. Although data are typically less readily available in low- and middle-income countries, available evidence suggests that the prevalence of non-adherence may be higher than in high-income countries, potentially due to barriers such as limited healthcare access, cost of medications and lower health literacy [4].

Medication non-adherence is commonly measured using methods such as the proportion of days covered (PDC) or medication possession ratio (MPR), metrics that typically use prescription issue data to assess the percentage of days within a specified time period that an individual has had access to their prescribed medication, although other newer techniques are described later in this review. When using assessment techniques such as these in the context of chronic diseases, adherence is typically defined as taking ≥80% of the prescribed doses of a given medication [5]. However, although adherence above this threshold has been associated with improved outcomes, it may be somewhat arbitrary, and the optimal threshold may depend on the specific condition or type of medication [2].

Adherence can also be conceptualised as occurring in three phases: initiation—failure to begin, or a delay in beginning, a newly prescribed treatment; implementation—regularly missing doses of a prescribed and initiated medication; and discontinuation—stopping taking a prescribed medication (or not being ‘persistent’) without first consulting a healthcare professional [6]. The related concept of medication persistence refers to the duration of time from treatment initiation to discontinuation [7]. While adherence includes both the regularity and timing of medication intake, persistence focuses solely on whether therapy is continued over time. Notably, an individual who is non-persistent would also be considered non-adherent, but the reverse may not always be true.

The objective of this narrative review is to provide a contemporary summary of the existing evidence relating to medication adherence in type 2 diabetes, including methods to assess adherence, factors relating to adherence, prevalence of non-adherence, consequences of non-adherence, and interventions designed to improve adherence in this population.

## Methods

This narrative review encompasses a broad, integrative overview of the literature, highlighting key themes and discussing conceptual and theoretical developments in the field. The author group, who supported and refined the content and presentation of the review, comprises an international panel of experts in the management of type 2 diabetes. Focused literature searches were conducted using combinations of the terms ‘type 2 diabetes’, ‘medication adherence’, ‘barriers’, ‘interventions’ and ‘outcomes’. Evidence from meta-analyses and systematic reviews was prioritised where available, with individual studies used to illustrate mechanisms or contextual factors. The aim was to provide a balanced review of the highest quality evidence available.

## Factors related to medication adherence

**Fig. 1:**
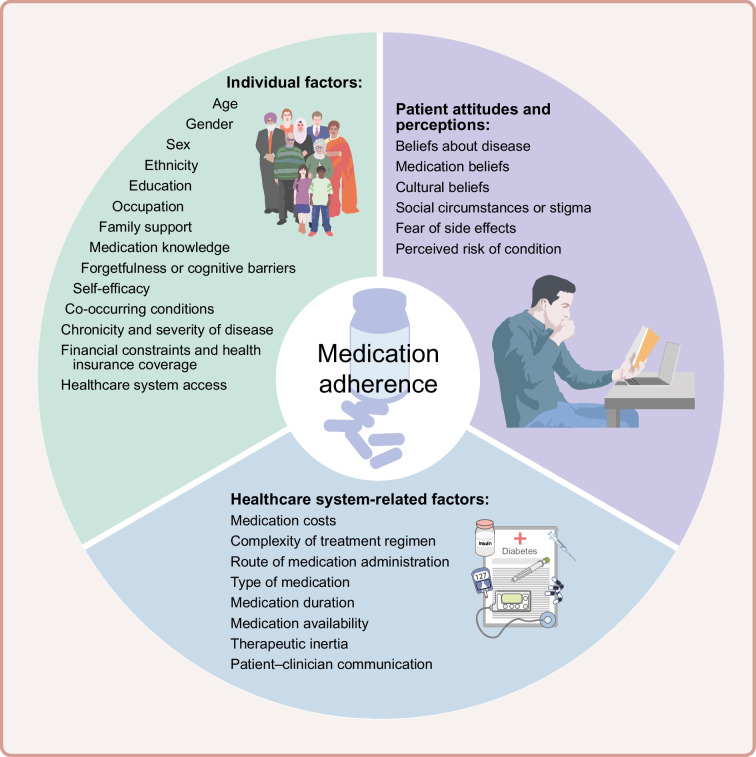
Factors influencing medication adherence in type 2 diabetes. This figure is available as part of a downloadable slideset

Medication adherence is a multifaceted issue that involves a complex interplay between individual, healthcare system-related and patient-related factors (Fig. 1) [1, 8, 9]:

- Individual factors include age, gender, sex, ethnicity, education, occupation, family support, medication knowledge, forgetfulness or cognitive barriers, self-efficacy, co-occurring conditions, chronicity and severity of disease, financial constraints, health insurance coverage and access to the healthcare system.
- Healthcare system-related factors include medication costs, complexity of the treatment regimen (e.g. polypharmacy or complex dosing frequencies), route of medication administration, type of medication, medication duration, availability of the prescribed medication, therapeutic inertia and level of communication between the patient and healthcare professionals.
- Patient attitudes and perceptions include beliefs about the disease and corresponding medications, cultural beliefs, social circumstances or stigma, fear of side effects, and perceived risk of the condition.

## Methods of assessing medication adherence

Medication adherence can be assessed using both subjective and objective methods [10]. Subjective measures include physician judgement of adherence status and self-reported adherence assessed using questionnaires or surveys (e.g. Morisky Medication Adherence Scale [MMAS]). These methods are inexpensive and simple to administer but may overestimate adherence due to recall or social desirability bias [11, 12], although only by 10–20% compared with objective methods [2]. However, self-reported methods have the advantage of identifying the reasons for non-adherence, which is vital for supporting intervention development to address non-adherence.

Objective methods can be categorised into those that directly assess medication-taking behaviours and those that measure medication taking indirectly (see Text box).


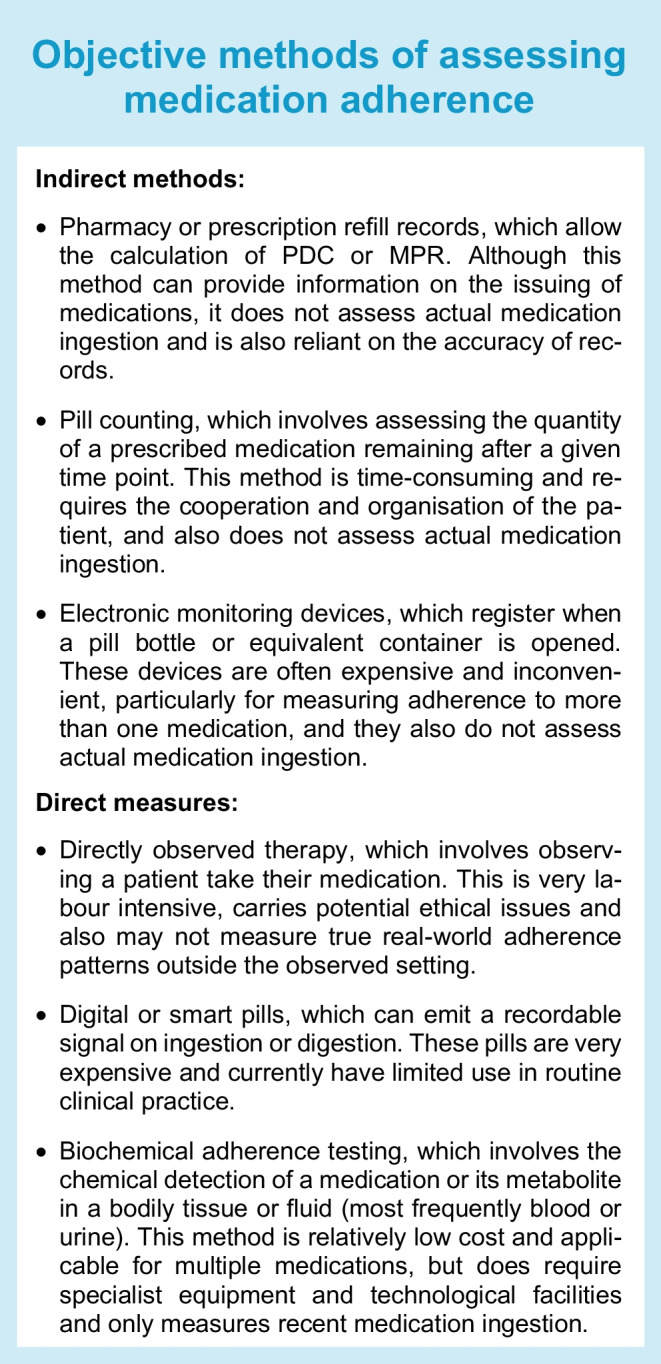


Objective measures of medication adherence are becoming more widespread in both research settings and clinical care, in particular biochemical adherence testing, which is now the preferred method of testing adherence in some clinical guidelines [13]. However, it should be noted that most measures of medication adherence, particularly direct measures when used in a research setting, are subject to the Hawthorne effect, a phenomenon in which individuals alter their behaviour because they are aware of being observed, which may artificially inflate adherence rates [14]. It should also be acknowledged that adherence typically varies over time, and single point assessments or assessments conducted over a short or arbitrary duration may not capture meaningful changes in long-term adherence that could influence health outcomes [15]. Ideally, adherence should be measured repeatedly, potentially combined with statistical methods to identify unique adherence patterns that may be overlooked by traditional methods [2].

There is currently no gold standard technique for measuring adherence, as all available methods have inherent limitations. Similarly, some techniques may be better suited to assess different domains of or reasons for non-adherence. For instance, electronic monitoring devices may be best suited to measuring adherence in those who are unintentionally non-adherent due to forgetfulness during the implementation phase of adherence; pharmacy records may best assess non-adherence due to cost or access barriers in the initiation or discontinuation phases of adherence; and self-report questionnaires or interviews may be best suited to investigate those who are intentionally non-adherent due to either their beliefs or their fear of side effects during any phase of adherence. In most cases, combining multiple methods, both subjective and objective, is preferable in order to assess the multiple reasons for and corresponding phases of non-adherence, tailored to clinical a priori knowledge about the population and medication(s) of interest.

## Medication adherence in type 2 diabetes

Globally, the prevalence of medication non-adherence in type 2 diabetes has been estimated to be 38% [16]; however, adherence varies by medication type, study population and method of assessment used. Adherence has been reported to be 63–68% for oral glucose-lowering drugs (OGLDs) [17, 18], 43–54% for injectable glucagon-like peptide-1 receptor agonists (GLP-1RAs) [19, 20] and 41–64% for insulin [21, 22]. Notably, another study reported that adherence rates for OGLDs are <50% in type 2 diabetes 1 year after initiation [23]. The prevalence of medication non-adherence in type 2 diabetes in the literature therefore varies greatly, likely due to variations in the operational definition of adherence employed, the different methods used to assess adherence, the phase of adherence assessed and the time frame over which adherence is assessed. For instance, people newly initiated to diabetes therapy have been shown to be 61% less likely to be adherent than those continuing therapy [24]. Furthermore, significant (1.6-fold) discrepancies in adherence to OGLDs have been observed when comparing objective vs subjective methods of assessment [25], and significant variations in adherence to GLP-1RAs have been observed when measuring continued adherence (67.9% adherent, 95% CI 59.6, 76.3) vs persistence (56.2%, 95% CI 46.1, 66.3) or discontinuation (31.4%, 95% CI 17.6, 45.3) [17].

The specific amounts and types of medications prescribed may also influence the prevalence of medication adherence. While an increase in the number of prescribed medications is generally associated with lower adherence [26], the reverse has been observed in diabetes, whereby adherence increases with each additional pill taken daily [24].

## Clinical and cost consequences associated with non-adherence in type 2 diabetes

Medication non-adherence is a significant challenge in the management of glucose levels in people with type 2 diabetes. It adversely affects multiple domains including glycaemic management, healthcare resource use and costs, quality of life, and risks of major adverse cardiovascular events and mortality.

### Glycaemic management

In people with type 2 diabetes, reduced adherence is associated with increased HbA_1c_ in a dose-dependent manner, with each 1 point increase in non-adherence on the MMAS-4 or MMAS-8 questionnaire associated with a 1.8 mmol/mol (0.16%) or 2.3 mmol/mol (0.21%) increase in HbA_1c_, respectively, after adjusting for baseline HbA_1c_ [27, 28]. In a retrospective cohort analysis, non-adherent individuals (MPR <80%) showed lower reductions in HbA_1c_ following glucose-lowering treatment than adherent individuals (−0.75%, 95% CI −0.78, −0.72 [−8.2 mmol/mol, 95% CI −8.5, −7.9] vs −1.14%, 95% CI −1.16, −1.13 [−12.5 mmol/mol, 95% CI −12.7, −12.4], respectively) [29]. Non-adherent individuals also had higher overall HbA_1c_ levels, diastolic BP and LDL-cholesterol levels than adherent individuals when based on PDC <80% (8.1% vs 7.7% [65 vs 61 mmol/mol], 75.8 vs 74.2 mmHg and 2.5 vs 2.2 mmol/l, respectively) [30] and reached LDL-cholesterol targets less often (67.4% vs 81.1%, respectively) when adherence was assessed using chemical adherence testing [31].

### Macrovascular and microvascular complications and mortality

In individuals with type 2 diabetes, reduced adherence (PDC) is associated with an increased risk of CVD events (cerebrovascular events and myocardial infarctions; HR 1.41 [95% CI 1.30, 1.52] for PDC of 20–39% vs ≥80%) [32]. Similarly, non-adherence to cardiometabolic medications in type 2 diabetes, identified via chemical adherence testing, has been associated with an increased risk of adverse cardiovascular outcomes (composite of myocardial infarction, stroke or CVD-related death; HR 10.13 [95% CI 3.06, 33.56] for non-adherence vs adherence to anti-platelet drugs) and renal outcomes (declining renal function, renal failure or renal-related death; HR 1.98 [95% CI 1.37, 2.86] for non-adherence vs adherence to BP-lowering medications) [33]. Adherent individuals (MPR ≥80%) have been shown to have a reduced risk of new microvascular or macrovascular diabetes complications (HR 0.96, 95% CI 0.92, 1.00) compared with non-adherent individuals [34].

The relationship between adherence and mortality risk in type 2 diabetes is particularly striking. Adherence to OGLDs has been associated with a 28% risk reduction in mortality (RR for adherent (MPR/PDC ≥80%) vs non-adherent: 0.72, 95% CI 0.62, 0.82) [16]. Greater reductions in risk have been observed with longer durations of adherence, with reported mortality RRs of 0.84 (95% CI 0.71, 0.98) and 0.69 (95% CI 0.57, 0.85) after 4–6 and ≥6 years of adherence (MPR ≥80%) to metformin, respectively [35]. Conversely, non-adherence to OGLDs (MPR <80%) in the first 2 years after prescription has been associated with a 40% greater risk of all-cause mortality (OR 1.40, 95% CI 1.01, 1.95) [36]. Other studies using large observational diabetes datasets have observed adjusted ORs or HRs for all-cause mortality of 1.45–1.81 when comparing non-adherent individuals (PDC <80%) with adherent individuals [30, 32].

Interestingly, the impact of adherence on mortality risk in type 2 diabetes may vary by ethnicity. A 5 year cohort study of ~630,000 US veterans found that the hazard of mortality for those in the lowest vs highest adherence (MPR) quintile varied by ethnic group: 12.21 (95% CI 11.89, 12.55) for non-Hispanic White participants, 10.01 (95% CI 9.18, 10.91) for non-Hispanic Black participants, 12.65 (95% CI 11.10, 14.43) for Hispanic participants and 10.41 (95% CI 9.06, 11.96) for other ethnicities [37]. This highlights both the significant impact of non-adherence on mortality risk in type 2 diabetes and the complexity of the relationship, which is likely to be related to a number of other clinical and sociodemographic factors.

Furthermore, an effectiveness gap has been identified between the efficacy of some novel organ-protective therapies (GLP-1RAs and dipeptidyl peptidase-4 inhibitors [DPP-4is]) in trial settings and their effectiveness in real-world scenarios. Individuals initiating these medications in the real world experience smaller reductions in HbA_1c_ than those participating in trials (−0.52% vs −1.30% [–6 mmol/mol vs –14 mmol/mol] for GLP-1RAs, −0.51% vs −0.68% [–6 mmol/mol vs –8 mmol/mol] for DPP-4is). Approximately three-quarters of this disparity is attributable to differences in medication adherence [38], with higher adherence in trial settings.

### Hospitalisation

A meta-analysis found that good adherence (primarily MPR/PDC ≥80%) to prescribed therapies in type 2 diabetes was associated with reduced risks of hospitalisation (HR 0.90, 95% CI 0.87, 0.94) and healthcare resource use [16]. Another study showed that each 1 point increase in non-adherence to basal insulin on the MMAS-8 questionnaire was associated with a 4.6% increase in number of physician visits, a 20.4% increase in number of emergency room visits and a 20.9% increase in number of hospitalisations [28]. A retrospective analysis found that adherence (PDC ≥80%) to glucose-lowering medications in people with diabetes lowered the odds of hospitalisation or an emergency department visit by 31%. This reduction increased to 53% among those adherent to glucose-, BP- and lipid-lowering medications [39].

### Quality of life

Medication adherence has been shown to have a significant and independent association with health-related quality of life (HRQoL) in people with type 2 diabetes. MMAS total score has been correlated with all HRQoL domains and the overall quality of life score (*r*=0.10–0.18 for the WHO Quality of Life-Brief questionnaire [40]; rho=0.136–0.230 for the EQ-5D-5L and EQ-VAS health questionnaires [41]). Similar findings have been reported in other studies [42, 43]. However, the direction of causality cannot be inferred here. It is possible that low medication adherence reduces quality of life through increased disease burden and adverse health outcomes. Conversely, low quality of life may impair an individual’s capacity to adhere to treatment regimens and engage in effective self-management, for instance if linked to depressive symptoms [43].

### Therapeutic inertia

Medication non-adherence can also indirectly impact outcomes in type 2 diabetes by driving therapeutic inertia. Evidence shows that people with type 2 diabetes in the lowest quartile of MPR-assessed adherence to hypoglycaemic therapies were less likely to have their treatments intensified within 12 months following an elevated HbA_1c_ result than those in the highest adherence quartile. In adjusted analyses, participants in the highest quartile of adherence had significantly greater odds of medication intensification (OR 1.53, 95% CI 1.11, 1.93) [44]. This highlights a potentially complex relationship between medication adherence and clinician willingness to adjust treatment regimens. Non-adherence may obscure the true cause of inadequate glycaemic management, leading clinicians to delay intensification, while high adherence may be interpreted as a signal that therapeutic changes are justified. Further research is needed to better understand this dynamic and its implications for timely, appropriate diabetes management.

### Costs associated with non-adherence

In type 2 diabetes, increased costs related to medication non-adherence are largely driven by elevated inpatient costs, which have been shown to be 41% higher in non-adherent (MPR <80%) individuals, while outpatient and pharmacy costs have been shown to be 7% and 37% lower in non-adherent individuals, suggesting that the financial burden of non-adherence lies in the avoidable downstream consequences [45]. This has been demonstrated in a study of insulin pen users between 2006 and 2010; individuals with the greatest insulin adherence (MPR) had higher overall annual pharmacy costs than those with the lowest insulin adherence (US$10,174 vs US$5395, respectively) [46]. This is unsurprising, as adherent individuals are more likely to engage with outpatient appointments (such as medication reviews) and incur pharmacy costs for medication dispensing; these costs would then be expected to be overwhelmingly offset by reduced healthcare costs as a result of medication non-adherence. This was confirmed in the same study: individuals in the least adherent group (MPR <20%) had greater overall healthcare costs per year than those in the most adherent group (MPR >80%; $26,310 vs $23,839, respectively) [46].

A study based on routine US healthcare data in type 2 diabetes found that adherence (PDC) of <20% vs ≥80% resulted in increased costs of US$28,824 over 3 years, while a 1% increase in adherence among 1000 individuals would save US$65,464 over 3 years (data from 2009–2014) [47]. In a larger cohort study of ~740,000 US veterans (data from 2002–2006), improving adherence in the non-adherent group was projected to result in annual estimated cost savings ranging from ∼US$661 million (MPR <0.6 vs ≥0.6) to ∼US$1.16 billion (MPR <1 vs 1) [45]. This supports the case for investment in adherence-enhancing strategies/interventions from both a clinical and an economic perspective.

## Barriers to medication adherence in type 2 diabetes

Several patient-, therapy- and system-related factors impede medication adherence in type 2 diabetes.

### Patient-related factors

Fear of hypoglycaemia remains a major concern among individuals with type 2 diabetes and is a leading cause of non-adherence [48, 49]. Psychological factors/comorbidities such as depression, anxiety and diabetes distress also contribute to non-adherence [37, 40]. Attitudes towards insulin also create barriers to adherence in type 2 diabetes. Barriers to the initiation of insulin include a desire to manage diabetes through lifestyle behaviours, a sense of personal failure, low self-efficacy for disease self-management and limited training [50], while individuals who have initiated insulin therapy report numerous barriers to adherence, including being too busy, travel disrupting insulin regimens, skipping meals, stress or emotional problems and public embarrassment [51]. Perceived treatment efficacy and perception of treatment risks can also be barriers to adherence in type 2 diabetes, along with other factors, such as forgetfulness driving unintentional medication non-adherence [52]. Those with physical limitations (e.g. poor eyesight due to diabetic retinopathy) or other physical disabilities also have greater barriers to adherence in type 2 diabetes [53]. Lastly, lack of social or family support may also impede efforts to be adherent to medication regimens in those with type 2 diabetes [54, 55].

### Therapy-related factors

Complex treatment regimens are a well-documented barrier to adherence. One study found that each increase in the number of antihypertensive medications led to a 77–85% increase in non-adherence, although this finding was observed outside a diabetes-specific population [26]. The PANORAMA study demonstrated that being on three or more oral or injectable glucose-lowering therapies was a significant predictor for not reaching glycaemic targets [56]. Having multiple long-term conditions (also known as multimorbidity) [57], which drives adverse outcomes in type 2 diabetes, particularly early-onset type 2 diabetes [58], also increases medication non-adherence, potentially because of the treatment burden induced by complex care needs [59].

### System-related factors

Effective communication between healthcare providers and patients also influences adherence rates; communication barriers and lack of shared decision making negatively impact adherence [52, 60], and reduced trust in physicians may also reduce medication adherence [52, 54]. High medication costs and limited healthcare or medication access are also significant determinants of non-adherence in diabetes [50, 61]. Provider knowledge regarding treatment intensification may also influence patient adherence status [62].

## Interventions to improve medication adherence

**Fig. 2:**
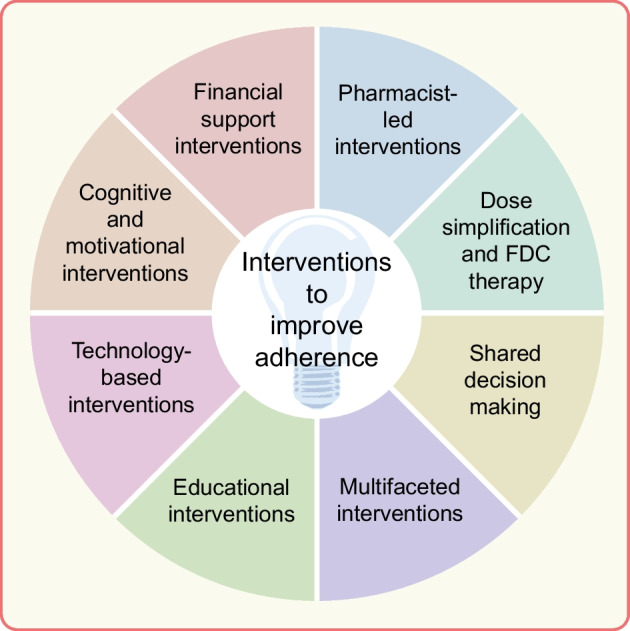
Intervention strategies to improve medication adherence in type 2 diabetes. This figure is available as part of a downloadable slideset

The WHO has stated that ‘Increasing the effectiveness of adherence interventions may have a far greater impact on the health of the population than any improvement in specific medical treatments’ [1]. This emphasises the importance of adherence-focused strategies in chronic disease management, particularly in type 2 diabetes. Various types of interventions have been tested to improve medication adherence, both generally and in type 2 diabetes [63, 64], and have shown mixed results depending on the intervention component investigated [65]. These components are discussed below and summarised in Fig. 2.

### Educational interventions

Patient education and empowerment through self-management programmes are a key component of interventions to improve medication adherence in type 2 diabetes [66] and several systematic reviews have investigated their effectiveness. While one review from 2008 showed little benefit due to lack of evidence (four eligible studies) [67], the field has since expanded substantially. More recent reviews have demonstrated positive effects of educational interventions on medication adherence in type 2 diabetes. These include interventions delivered by pharmacists or nurses, individual education sessions, group-based education programmes, and education delivered at home or in a healthcare setting [68, 69], although data heterogeneity has precluded meta-analyses in some cases.

The effectiveness of these interventions may be mediated through increased diabetes-related self-efficacy (i.e. an individual’s confidence in their ability to manage diabetes effectively), which can be measured using questionnaires such as the Diabetes Management Self-Efficacy Scale [70]. Educational interventions can increase diabetes-related self-efficacy in type 2 diabetes [71] and have been shown to be significantly associated with medication adherence (β=0.53 and *r*=0.352 in two studies, respectively [72, 73]). In cardiovascular medicine, interactive educational interventions delivered in person by a clinician show greater effects than non-interactive interventions delivered remotely [63]; however, online educational interventions have also shown benefits in diabetes [74] and may be more appropriate in resource-limited settings. Education can also be delivered via app-enabled interventions, which have been shown to be effective in improving adherence over 3 months in type 2 diabetes compared with usual care (standardised mean difference [SMD]: 0.393, 95% CI 0.17, 0.61) [75]. Lastly, a recent systematic review of 55 studies identified that educational interventions that address medication adherence only (i.e. not focusing more widely on disease self-management) and are delivered by pharmacists are the most effective, along with those that incorporate specific behaviour change techniques [76].

Healthcare professionals may also benefit from receiving education, for instance education regarding available tools and methods for identifying medication non-adherence, and skills required to effectively manage and support non-adherent individuals [77]. Educational interventions can be underpinned by behaviour change theories or frameworks. For instance, the COM-B (capability, opportunity, motivation–behaviour) model [78], which posits that for an individual to engage in a specific behaviour (such as being adherent to a medication regimen) they must possess the mental and physical capability, and have the necessary opportunity and motivation, has been used to investigate enablers of and barriers to medication adherence in type 2 diabetes [79] and may be used to support medication adherence interventions [80]. Similarly, the perceptions and practicalities approach (PaPa) conceptualises medication adherence by examining how a person’s perceptions (motivation) and practicalities (ability) interact with external factors such as opportunities (e.g. access to treatment) and triggers (e.g. prompts to action) to determine the likelihood of being adherent [81]. This approach may therefore also be used to support the design of interventions that address these factors.

### Technology-based interventions

Technology-based interventions, such as SMS reminders and mobile apps, have shown varied success in improving medication adherence in type 2 diabetes. A systematic review and meta-analysis from 2016 found little evidence of benefit compared with control of either brief messages alone or messages combined with medication monitoring in improving medication adherence in type 2 diabetes (five studies; SMD 0.22, 95% CI −0.05, 0.49) [82]. A more recent systematic review of mobile phone text message reminders in type 2 diabetes showed evidence of benefit compared with usual care (nine studies; SMD 0.36, 95% CI 0.14, 0.59); however, the effects were greatest with intervention durations of more than 6 months [83]. In addition to serving as simple reminders, messages can also be tailored and designed to improve disease self-management or target various domains of behaviour change in order to improve adherence [84, 85].

Mobile phone-based apps can also be used to improve medication adherence in type 2 diabetes, for instance by providing medication reminders, adherence or test result monitoring, or educational information on disease and lifestyle management (as described above); facilitating interactions with clinicians or shared decision making; or providing cloud-based functionality and gamification of disease and adherence management [75, 86]. A recent systematic review that included app-based interventions for type 2 diabetes comprising four main features (encouraging self-monitoring of glucose, medication reminders, patient education or healthcare professional support) found that they were effective at improving medication adherence (meta-analysis not conducted) and reducing HbA_1c_ (six studies; mean reduction: 0.36%, 95% CI 0.25, 0.47 [3.9 mmol/mol, 95% CI 2.7, 5.1]) compared with control [86]. Another systematic review found similar effects of mobile apps on medication adherence compared with control (five studies; mean HbA_1c_ reduction: 0.664%, 95% CI 0.506, 0.823 [7.3 mmol/mol, 95% CI 5.5, 9.0]) [87], and a systematic review of seven studies also found adherence benefits in individuals with type 2 diabetes following app-based interventions, although again meta-analyses were not conducted because of outcome heterogeneity [88].

Conversely, a systematic review of 13 studies found that mobile health interventions did not improve medication adherence in those with type 2 diabetes, hypertension and/or dyslipidaemia, while telehealth interventions did [89]. A subsequent systematic review of 18 studies confirmed that telehealth interventions significantly increased medication adherence in those with type 2 diabetes (SMD 0.501, 95% CI 0.231, 0.771) [90]. Gamification is another recent technology-enabled strategy used to target motivation and incentivisation to improve diabetes self-management and glycaemic management [91], although its impact on medication adherence in diabetes is currently limited and requires further research [92].

While heterogeneity in study and systematic review design in this burgeoning field makes comparisons difficult, this is a promising area of research with strong evidence for benefit in improving medication adherence in type 2 diabetes, particularly given the post-COVID-19 landscape of healthcare delivery and the availability of telehealth services. However, efforts should also be made to reduce the digital divide and prevent strengthening of existing health inequalities regarding diabetes management when considering technology-based interventions. One study identified disparities in the use of a mobile health intervention to promote medication adherence in low-income adults with type 2 diabetes, with those from ethnic minority communities, older adults and those with lower health literacy or more depressive symptoms less likely to be engaged in the intervention [93]. Furthermore, technology-based interventions may be particularly suitable for younger individuals with type 2 diabetes who are able to engage with smartphones, while older adults or those with cognitive impairment may face greater barriers to accessing or engaging with this type of intervention.

### Cognitive and motivational interventions

Addressing motivation to take medicines may be effective in improving medication adherence in type 2 diabetes. Motivational interviewing may be a suitable approach and has shown promising (although inconsistent) results in improving medication adherence generally in chronic conditions [63, 94]; however, the existing evidence specifically for diabetes is limited. One wider systematic review included six studies of motivational interviewing in those with endocrine conditions, only two of which found any improvement in medication adherence in the intervention group [95]; two other reviews that specifically investigated the impact of motivational interviewing in those with type 2 diabetes either did not identify any studies that evaluated medication-taking behaviour [96] or found no effect on medication adherence where measured [97].

Other cognitive-based interventions, such as cognitive behavioural therapy (CBT), can support diabetes care by targeting issues such as distress or depression, illness beliefs or self-management behaviours. CBT is a frequently used psychological intervention for supporting type 2 diabetes management and can be combined with behaviour change techniques such as social support, problem solving and goal setting [98]. A meta-analysis of 19 studies found CBT to be effective in reducing HbA_1c_ compared with usual care in those with diabetes when treatment duration was at least 6 months (SMD −0.44, 95% CI −0.63, −0.25) [99].

CBT-based interventions have also been shown to increase medication adherence in those with type 2 diabetes. Two RCTs in those with type 2 diabetes and depression showed small to moderate improvements in medication adherence following CBT-based interventions of similar durations (~4 months) and intensities (9–12 sessions) compared with enhanced usual care (one CBT session) or waitlist control groups [100, 101]. However, another study in those with type 2 diabetes and regimen-related distress found no impact of CBT on medication adherence compared with usual care [102]. CBT-based interventions may be most effective in individuals with type 2 diabetes and coexisting depression or other mental health conditions, potentially enhancing adherence either directly through behavioural support or indirectly by improving mental health. However, certain barriers exist that may limit CBT effectiveness, such as the necessity for long-term engagement, sustainability, and potential barriers related to cognitive impairments commonly observed in older adults with type 2 diabetes [103].

### Financial support interventions

Financial support interventions have been shown to improve all phases of medication adherence when investigating cardiovascular and cancer medications [104]. In a systematic review of 38 studies (eight on medication adherence, conducted primarily in the USA), lower co-payments were associated with better adherence in seven studies [105], highlighting financial burden as a potential barrier. A systematic review of 22 studies (21 of which were conducted in the USA) evaluated the effect of financial incentives on medication adherence in type 2 diabetes (vs no financial incentives); 16 studies demonstrated a positive effect, which meta-analyses revealed to be statistically significant but potentially not clinically meaningful (SMD 0.02–0.03 depending on outcome and subgroup) [106]. Another systematic review identified five studies (four in the USA, one in Canada) that investigated the influence of financial incentives on the behaviour of participants with diabetes, including medication adherence, all of which demonstrated a significant positive impact [107]. However, data regarding the impact of financial incentives on clinical outcomes in diabetes are mixed [107, 108]. This area of research is particularly relevant in settings without universal healthcare coverage, where the cost of acquiring medications is a major barrier to adherence. In contrast, in systems with subsidised care, financial incentives may play a less prominent role. Additionally, most research has been conducted in the USA or other high-income countries (such as Canada or Japan), which may limit the relevance of such interventions to middle- or low-income countries. Similarly, the scalability and sustainability of these types of interventions warrants further investigation.

### Pharmacy-led interventions

As the landscape of healthcare delivery and management changes, the roles of other members of the healthcare team are expanding. A key example of this is the role of pharmacists, which has shifted from delivering medication and dispensing services only to a more holistic approach to patient care and well-being, forming a key part of the multidisciplinary care team responsible for delivering diabetes care [109]. Pharmacists can deliver a variety of services to support improved medication adherence in diabetes, including patient education, medication reviews, multimodal reminders or organisational support and patient monitoring [110]. Earlier studies showed inconsistent findings [110], but more recent meta-analyses suggest that pharmacist-led interventions can significantly improve both medication adherence and glycaemic management in people with type 2 diabetes [111, 112].

Improvements in adherence following pharmacist-led interventions in diabetes have also been shown to lead to improved HbA_1c_ levels and glycaemic management [113, 114], and numerous systematic reviews in other conditions (such as CVD) have also observed benefits of pharmacist-led interventions in improving adherence and outcomes [115, 116], although, as is common in systematic reviews of medication adherence, outcome heterogeneity often precludes the completion of meta-analyses. Strategies to increase medication adherence in diabetes can also be embedded in wider disease self-management interventions delivered by pharmacists to improve overall risk factor management [117]. Furthermore, the expanding role of community pharmacies allows for greater ease of access to patient services, particularly in deprived areas where barriers to accessing care can be numerous and significant. For instance, in England, 99.8% of individuals living in the most deprived areas live within a 20 min walk of a community pharmacy [118]. Lastly, in the UK, all newly qualified pharmacists from 2026 will become independent prescribers on registration, further expanding their role in medication management.

### Dose simplification and fixed-dose combination therapy

The simplification of dosing regimens in diabetes management can improve medication adherence [119, 120]. Treatment complexity is a significant barrier to medication adherence, and adherence decreases in a dose-dependent manner with increasing number of prescribed doses per day [121]. One promising strategy to improve adherence in type 2 diabetes is the use of fixed-dose combination (FDC) or single-pill combination therapy, that is, combining two or more active drugs into a single dose. FDC therapy simplifies treatment by reducing the pill burden while maintaining the bioavailability of each individual medication [122], which has been shown to enhance adherence.

A meta-analysis of 61 studies of individuals with chronic disease (i.e. not type 2 diabetes specific) demonstrated that those receiving FDC therapy had significantly higher adherence rates than those receiving free-equivalent combination therapy (RR of adherence, intervention vs control: 1.29, 95% CI 1.23, 1.35) [123]. FDC therapy may also improve persistence in type 2 diabetes compared with free-equivalent combination therapy, supporting greater adherence over time [124]. In a meta-analysis of ten studies comparing FDC therapy with co-administered dual therapy using glucose-lowering agents, FDC therapy displayed greater effectiveness in both improving adherence and reducing HbA_1c_ (mean difference in HbA_1c_: −0.53%, 95% CI −0.78, –0.28 [–5.8 mmol/mol, 95% CI –8.5, –3.1]) [119].

Real-world studies confirm these benefits. Cheong et al reported that individuals with diabetes who switched from dual therapy to FDC therapy showed a 12.4% increase in adherence (measured via MPR), and that FDC therapy users were significantly more likely to demonstrate higher adherence than dual-therapy users (OR 1.867, 95% CI 1.716, 2.032) [120]. Phung et al found that individuals on early combination therapy with metformin achieved lower HbA_1c_ levels than those on monotherapy (weighted mean difference: −0.43%, 95% CI −0.56, −0.30 [–4.7 mmol/mol, 95% CI –6.1, –3.3]) [125]. FDC therapies have also been shown to provide cardiovascular benefits. Combining sodium–glucose cotransporter 2 (SGLT-2) inhibitors with metformin improves glycaemic management while reducing cardiovascular risk [126]. In analyses of real-world data, FDC therapies have been linked to improved adherence compared with multiple-pill therapies (59.9% vs 26.9% adherent), a lower incidence of cardiovascular events and death (105.8 vs 139.0 per 1000 person-years) and reduced health resource use (average annual direct healthcare costs of €2970 vs €3642 between 2010 and 2020) [127]. As such, FDC therapy provides a promising avenue for improving adherence and clinical and cost outcomes in type 2 diabetes, particularly as more novel therapies such as SGLT-2 inhibitors can be used in combination therapy to improve the management of glucose levels, metabolic control and cardiovascular outcomes while minimising the risk of hypoglycaemia and drug–drug interactions [126].

### Shared decision making

Shared decision making is the process of involving patients in disease treatment and management decisions in a collaborative effort with their clinicians [128]. As physician–patient communication is vital for improving adherence, it is important for healthcare professionals to implement shared decision-making strategies to foster trust and encourage adherence [52, 60]. One study in type 2 diabetes found that shared decision making was associated with improvements in participant activation, which in turn was associated with improved medication adherence and HbA_1c_ and LDL-cholesterol levels [129], although a meta-analysis of 16 studies in type 2 diabetes found little evidence of an association between shared decision making and medication adherence or glycaemic management [130]. Similar findings were observed in another, non-diabetes-specific systematic review of 23 studies, which reported moderate evidence for no effect of shared decision making on medication adherence [131].

### Multifaceted interventions

Given the complexity of adherence behaviour, multifaceted interventions are likely to be more effective than single-component strategies, as they can address multiple interacting barriers simultaneously [64, 132]. A study of a multifaceted intervention combining disease self-management education sessions and medication management support (regular phone calls to review adherence, monitor test results and make changes to prescriptions) reported greater odds of self-reported medication adherence in the intervention group compared with the usual care control group (OR 4.4, 95% CI 1.8, 10.6) [133]. Another study in people with uncontrolled type 2 diabetes (HbA_1c_ >53 mmol/mol [7%]) implemented a multifaceted intervention that comprised pharmacist-led education on medication adherence, provision of a diabetes diary log for recording medication details and other lifestyle factors and provision of a pill box. Following the intervention, 44.4% of the intervention group achieved the target HbA_1c_ level (<53 mmol/mol [7%]) compared with only 3.4% in the control group [134], highlighting the potential of multifaceted adherence interventions to improve glycaemic management in type 2 diabetes.

Multicomponent interventions may be most effective when delivered over a longer duration (>10 months) to support sustained improvements in medication adherence [135]. Future research should examine the potential of tailoring intervention component(s) to specific patient needs and barriers to directly target the reasons for non-adherence, and aim to identify which combinations of components are most effective. A network meta-analysis highlighted that interventions that comprise educational, attitudinal and technical components were particularly effective [135]. This is important in resource-limited areas where implementation of multiple intervention components may be challenging.

Lastly, the role and input of the prescriber are vital, regardless of the type of adherence intervention delivered or mode of delivery. Ongoing training based on contemporary guidelines on glucose-lowering medications and their use in specific patient populations is key to ensuring that prescribers provide up-to-date information to patients when discussing medications. For instance, advances in precision medicine may enable clinicians to identify those at greater risk of adverse events or side effects, or those most likely to respond positively to a given medication [136]. Such approaches could be powerful tools for supporting long-term adherence to therapies, although this is a relatively new area of research.

## Limitations of this review

As this was a narrative review, a systematic search was not conducted, which may have introduced subjectivity or bias in both the selection and the reporting of studies. Given the broad scope of this review, including the prevalence of non-adherence, barriers to adherence, outcomes of non-adherence and interventions to improve adherence, a narrative approach was chosen to provide a comprehensive overview of each area. Regardless, we acknowledge that this is not an exhaustive summary of all relevant publications in each area.

## Conclusion

Medication non-adherence remains a significant challenge in the management of type 2 diabetes and contributes to poor clinical and economic outcomes. How adherence is measured is also key; future research should aim to use a mixed-methods approach comprising both subjective and objective measures, aligning the choice of methods with the suspected or likely reasons for non-adherence in a given population or for a specific medication. Recognition should also be given to the fact that adherence varies over time and so one-off measurements of adherence may not tell the full story for a given individual.

Numerous evidence-based interventions exist to improve medication adherence in type 2 diabetes, including educational, technology-based, financial incentive-based, pharmacy-led, dose simplification and FDC therapy interventions, while more limited evidence supports the use of cognitive or motivational interventions and shared decision making. Interventions may be most effective when tailored to specific individuals and their barriers to medication adherence and when delivered over a minimum period of 3 months. FDC therapy interventions have particular promise by reducing the medication burden and combining traditional glucose-lowering therapies with newer organ-protecting therapies, although they do not address common barriers to adherence seen with injectable therapies. Importantly, multifaceted interventions appear to be effective, as patients often face multiple barriers to medication adherence. This is particularly relevant in type 2 diabetes, where treatment typically involves multiple medications delivered by different modes (oral and injectable), each associated with different barriers. Therefore, future research should prioritise the development of personalised, multicomponent interventions, delivered over at least 3 months and tailored to specific barriers to adherence in the target population, with adherence measured using a combination of objective and subjective methods.

## Supplementary Information

Below is the link to the electronic supplementary material.

Slideset of figures (PPTX 319 KB)

## Data Availability

No new data were created or analysed in this article. Data sharing is not applicable to this article.

## Abbreviations

CBT: Cognitive behavioural therapy
FDC: Fixed-dose combination
GLP-1RA: Glucagon-like peptide-1 receptor agonist
MMAS: Morisky Medication Adherence Scale
MPR: Medication possession ratio
OGLD: Oral glucose-lowering drug
PDC: Proportion of days covered
SGLT-2: Sodium–glucose cotransporter 2
SMD: Standardised mean difference

## Authors’ relationships and activities

FZ has acted as a consultant for Servier, Menarini and Daiichi Sankyo. L-LL has received research grants via her institution from Abbott Diabetes Care, AstraZeneca and Novartis; and speaker honoraria from Abbott, AstraZeneca, Boehringer Ingelheim, Novo Nordisk, Roche Diabetes Care and Zuellig Pharma. SS has received grants from Sanofi, AstraZeneca, Novo Nordisk, Boehringer Ingelheim, Servier and Lilly; speaker fees from Boehringer Ingelheim, Lilly, MSD, Novartis, Novo Nordisk, Janssen, Amgen, Sanofi, AstraZeneca, Abbott, Menarini, Dexcom and Daiichi Sankyo; travel support from AstraZeneca, Abbott, Menarini and Dexcom; and advisory board fees from Boehringer Ingelheim, Lilly, Novo Nordisk, AstraZeneca, Abbott and Dexcom. KK has acted as a consultant or speaker for, or received grants for investigator-initiated studies from, AstraZeneca, Bayer, Novartis, Novo Nordisk, Sanofi-Aventis, Lilly, Merck Sharp & Dohme, Boehringer Ingelheim, Oramed Pharmaceuticals, Pfizer, Roche, Daiichi Sankyo and Applied Therapeutics. The authors declare that there are no other relationships or activities that might bias, or be perceived to bias, their work.
