# Supplementary material for: Improving medication adherence in type 2 diabetes: strategies for better clinical and economic outcomes
Source: Diabetologia. 2025 Nov 28;69(3):541–56. doi: 10.1007/s00125-025-06617-x (PMC12881013; doi:10.1007/s00125-025-06617-x)
Supplement: Supplementary file 1 — Slideset of figures (PPTX 319 KB) [file 125_2025_6617_MOESM1_ESM.pptx]

## Slide 1
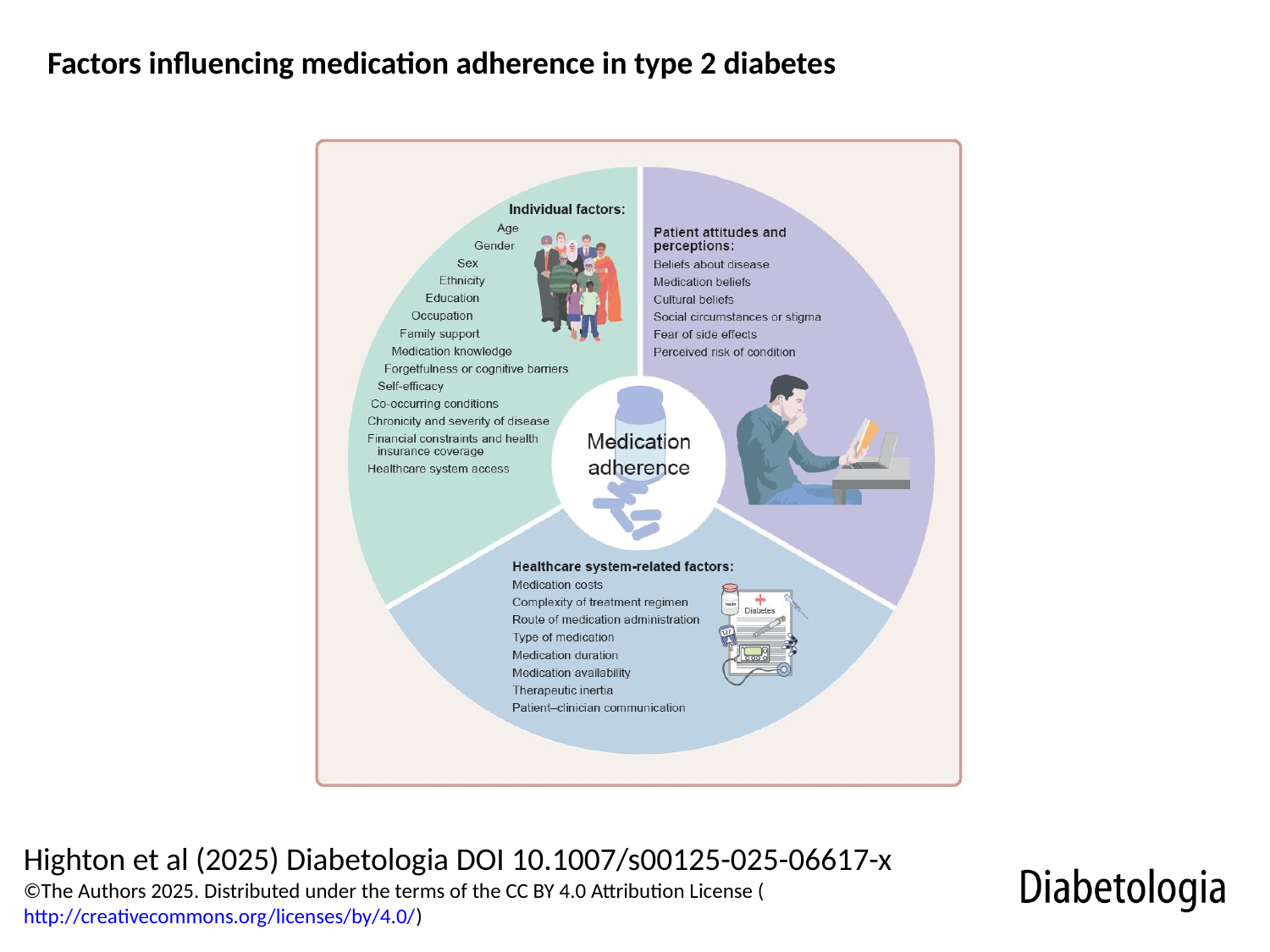

Factors influencing medication adherence in type 2 diabetes
Highton et al (2025) Diabetologia DOI 10.1007/s00125-025-06617-x
©The Authors 2025. Distributed under the terms of the CC BY 4.0 Attribution License (http://creativecommons.org/licenses/by/4.0/)

## Slide 2
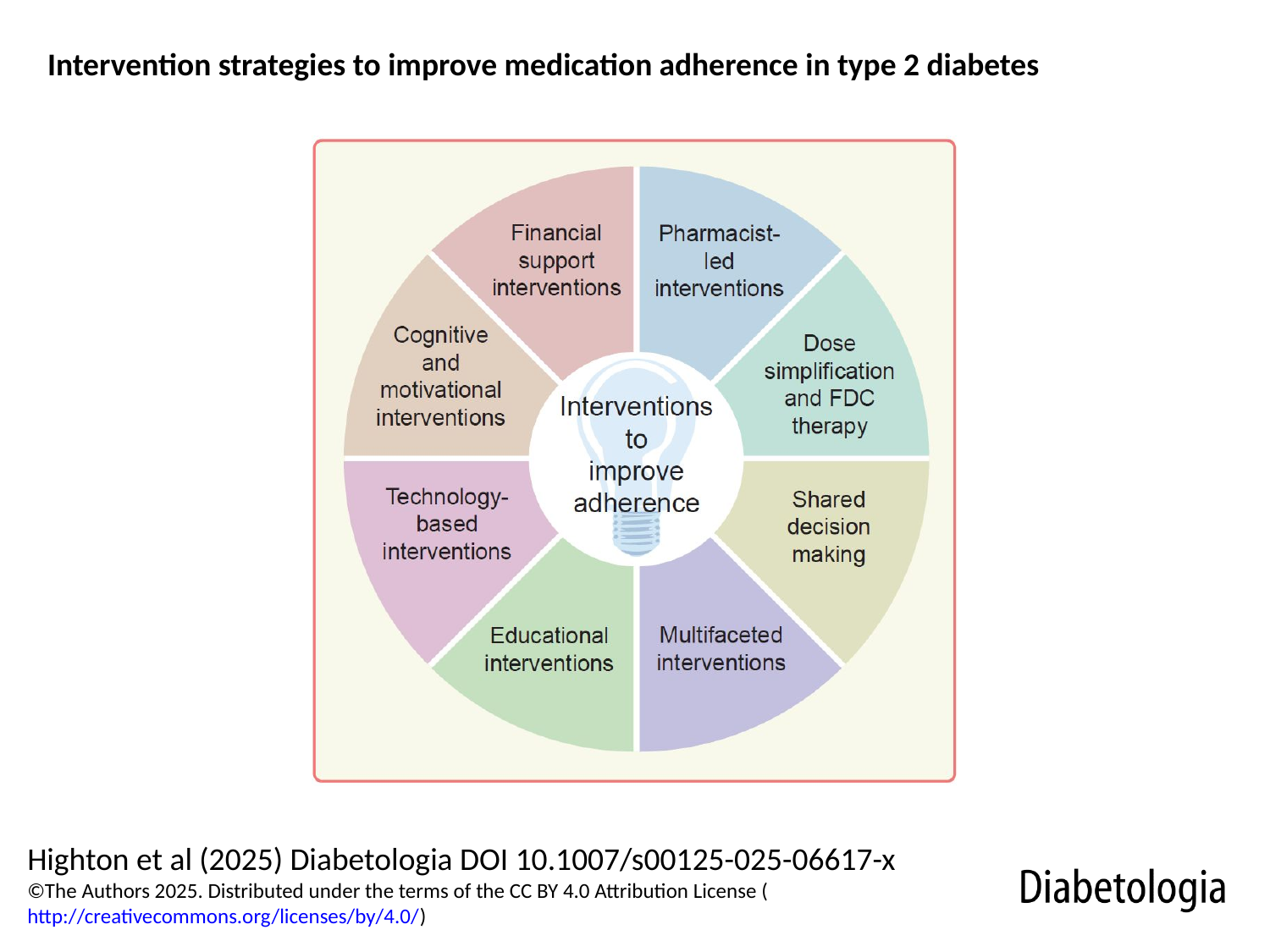

Intervention strategies to improve medication adherence in type 2 diabetes
Highton et al (2025) Diabetologia DOI 10.1007/s00125-025-06617-x
©The Authors 2025. Distributed under the terms of the CC BY 4.0 Attribution License (http://creativecommons.org/licenses/by/4.0/)
